# Association of Preoperative Optical Coherence Tomography Markers with Residual Inner Limiting Membrane in Epiretinal Membrane Peeling

**DOI:** 10.1371/journal.pone.0066217

**Published:** 2013-06-11

**Authors:** Gerald Seidel, Martin Weger, Lisa Stadlmüller, Tamara Pichler, Anton Haas

**Affiliations:** 1 Department of Ophthalmology, Medical University of Graz, Graz, Austria; 2 Institute for Medical Informatics, Statistics and Documentation, Medical University of Graz, Graz, Austria; Medical University Graz, Austria

## Abstract

**Purpose:**

To identify preoperative markers on spectral domain optical coherence tomography (SD-OCT) for residual inner limiting membrane (ILM) in epiretinal membrane (ERM) peeling.

**Methods:**

In this retrospective case series the preoperative SD-OCTs from 119 eyes of 119 consecutive patients who underwent surgery for idiopathic ERM by a single surgeon were evaluated for markers predisposing for ILM persistence after ERM removal. ILM persistence was determined via intraoperative indocyanine green staining. The main outcome measures were correlation of central foveal thickness (CFT), ERM thickness, extent of elevated ERM and retinal folding, intraretinal cysts, and discontinuation of the ERM, with ILM persistence after ERM peeling.

**Results:**

The persistence of the ILM was found in 50.4% (n = 60). After Bonferroni correction for multiple testing, a greater extent of elevated ERM and thicker ERMs were associated with persistence of the ILM (p<0.005). The other parameters showed no statistically significant correlations with the persistence of the ILM (p≥0.005).

**Conclusion:**

Residual ILM can be found in nearly half of the eyes after ERM peeling. A loose connection between the ERM and the retinal surface predisposes for ILM persistence. Preoperative SD-OCT is helpful in identifying risk markers for the persistence of the ILM in ERM surgery.

## Introduction

Epiretinal membranes (ERMs) are avascular tissue sheets that grow on the retinal surface. They consist to varying degrees of glial cells, hyalocytes, fibroblast-like cells, and retinal pigment epithelial cells.[1], [2] ERMs can contract, causing disturbance of the retinal architecture, which may subsequently lead to metamorphopsia and decreased central visual acuity.

The inner limiting membrane (ILM) serves as a scaffold for ERM growth and it has been shown that after removal of only the ERM, glial cells, hyalocytes, and myofibroblasts remain on the ILM.[3] This is thought to be a major factor for ERM recurrences. Recurrence rates of up to 56% have been reported in patients with the ILM left on the retina compared to 9% after additional ILM removal.[4], [5], [6], [7] Thus, when treating ERMs via membrane peeling adjunct removal of the ILM has been advocated.

The ILM, however, remains on the retina in between 50.8 to 64% of cases after ERM peeling.[3], [8] In these cases a second peeling process to remove the ILM is needed. This study demonstrates predisposing markers on preoperative high-resolution spectral domain optical coherence tomography (SD-OCT) for the persistence of the ILM after ERM removal.

## Methods

A retrospective chart evaluation of all patients undergoing ERM peeling at the Medical University of Graz from August 2010 to November 2011 was performed. Exclusion criteria were any previous ocular surgery other than phacoemulsification, a history of uveitis, diabetic retinopathy or maculopathy, macular hole, myopia of more than 6 diopters, lamellar hole, age-related macular degeneration, and history of ocular trauma. One hundred twenty one eyes from 119 consecutive patients with idiopathic ERM met these criteria. From two patients who underwent surgery on both eyes only the first eyes were included, leading to 119 eyes overall. The mean age was 72±8 years, 53% (n = 63) were women, and mean best-corrected visual acuity was logMAR 0.5±0.24 (decimal equivalent of 0.32 median). The study was conducted according to the declaration of Helsinki and was approved by the ethics committee of the Medical University of Graz. All data were collected as part of routine diagnostics and treatment. All patients were diagnosed and treated according to national guidelines and agreements. Data analysis was performed anonymously and retrospective. The authors state that individual patient consent was not obtained and hereby confirm that the ethics committee of the Medical University of Graz agreed that individual patient consent was not needed for this study.

All patients underwent 3-port 23-gauge vitrectomy (OS3; Oertli, Berneck, Switzerland) with intraoperative indocyanine green (ICG) staining of the ILM (0.2% solution; ICG-Pulsion, Pulsion Medical Systems AG, Munich, Germany) before and after ERM removal. A single experienced surgeon (A.H.) conducted the surgery in all patients to ensure reproducibility. To maximize the rate of combined removal of the ILM and ERM the peeling was initiated by grasping the stained ILM at the border of the ERM. After primary peeling the macula area was stained a second time with ICG to visualize residual ILM. Any persistence of ILM in the previously peeled area was defined as residual ILM and peeled in a second step. These cases were termed separate peeling cases, in contrast to combined peeling in case of simultaneous removal of the ERM and ILM. To minimize the potential retinotoxic effect of ICG meticulous attention towards iso-osmolality and minimization of the duration as well as the area of exposure was paid.[9], [10] Thus, the dye was washed out immediately after its application on the retina in a fluid filled eye.

SD-OCTs via the 5 line mode of the Cirrus 4000 OCT (Carl Zeiss Meditec, Inc.) were obtained one day before surgery and 7 parameters were assessed: central foveal thickness (CFT), ERM thickness (ERM-Th), ERM elevation, retinal folding, discontinuation of the ERM, and intraretinal cysts. Because of potential erroneous detection by the OCT’s integrated algorithms, all parameter were assessed manually by means of the device’s inbuilt caliper tool. One technician took all OCTs and two retina specialist performed the parameter assessment. The OCT investigators were not involved in the surgery and blinded regarding intraoperative ILM persistence after ERM peeling.

**Figure 1:**
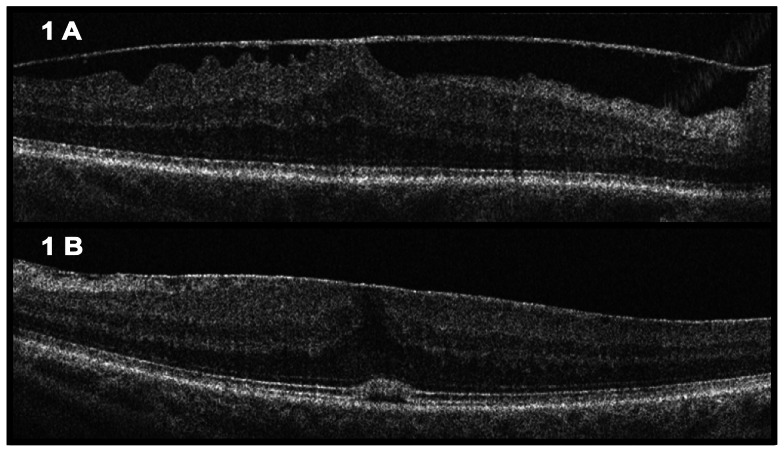
Optical coherence tomography of two eyes with dissimilar characteristics of the epiretinal membrane (ERM): 1A shows almost complete elevation of the ERM and retinal folds in the temporal segment; 1B shows a diffusely adherent ERM to the retinal surface without elevation and minimal nasal folding.

All data were obtained from the horizontal 6 mm OCT section centered at the fovea. ERM-Th was defined as the thickest part of the ERM within these bounds. Measurement was taken after appropriate zooming. Any non reflective space between the ERM and ILM was defined as ERM elevation. All segments within one scan depicting this feature were added up, consequently leading to a potential distance range from 0 to 6 mm. Retinal folds were assessed in a similar fashion. Any interruption in the ERM was defined as discontinuation of the ERM. Any cystoid space in the retina was counted positive for retinal cysts. Figure 1 shows two examples of OCTs depicting dissimilar characteristics.

With respect to normal distribution, we used the unpaired t-test or the Mann-Whitney-U test to compare continuous variables. To test categorical variables we used the exact Fisher test and the Pearson chi-quadrat test. Bonferroni correction was used to adjust for multiple testing. Subsequently, a p-value of <0.005 was considered statistically significant. Analysis was performed with SPSS (Version 20.0 for Windows, SPSS, Inc., Chicago, IL).

## Results

The mean age was 72.6±8.4 years and 53% were women. The mean BCVA was 0.5±0.21 logMAR (decimal median 0.32). All 119 eyes underwent uncomplicated ERM peeling. Repeating the ICG staining after complete ERM removal showed persistence of the ILM in 60 out of 119 eyes (50.4%).

To detect predictive factors for combined or separate removal of the ERM and ILM we tested for the following parameters: age, sex, preoperative BCVA, lens status, intraoperative vitreoretinal status, CFT, ERM-Th, ERM elevation, retinal folding, discontinuation of the ERM, and intraretinal cysts.

Sex, age and preoperative BCVA were not correlated with ILM persistence (p > 0.005; data not shown). Out of the 99 phakic patients included into the study 95 had concomitant phacoemulsification and intraocular lens implantation. The status of the lens did not affect ILM peeling (p = 0.626). The vitreous presented intraoperatively detached in 79.8% (n = 95), incompletely detached in 3.3% (n = 4), and completely attached in 16.8% (n = 20) of the cases. The vitreal status did not differ between the combined and the separate peeling group (p = 0.81).

On OCT the mean distance of retinal folding was higher in the separate – than in the combined peeling group (2.5±1.8 and 1.7±1.6, respectively), however, without reaching statistical significance after Bonferroni correction (p = 0.008). The mean distance of ERM elevated from the retinal surface was significantly longer in the separate peeling group (1.5±1.5 versus 0.7±1.1 mm; p = 0.001).

ERM-Th was statistically significant higher in the separate peeling (p = 0.002). The mean ERM-Th was 18.9±10.8 µm in cases with persisting ILM and 13.8±3.3 µm in the combined peeling group. A trend towards higher CFT in patients with ILM persistence than in the combined peeling group was observed (511.4±113 and 473.3±102, respectively; p = 0.028). The presence of intraretinal cysts did not differ between the two groups (p = 0.632). There was a trend towards discontinuation of the ERM in the separate peeling group (31.7% vs. 15.3%), yet without reaching statistical significance after Bonferroni correction (p = 0.032). Table 1 shows the morphologic parameters for the separate peeling – and for the combined peeling group.

**Table 1:** Characteristics of eyes undergoing epiretinal membrane (ERM) peeling.

	Combined peelingn = 59 (49.6%)	Separate peelingn = 60 (50.4%)	P value*
Vitreal status (n (%))			0.801
*attached*	8 (13.6%)	12 (20.0%)	
*incompletely detached*	2 (3.4%)	2 (3.4%)	
*detached*	49 (83.0%)	45 (76.7%)	
Central foveal thickness† (µm)	473.3±101.8	511.4±113.1	0.028
ERM thickness† (µm)	13.8±3.3	18.9±10.8	0.002
Retinal folding† (mm)	1.7±1.6	2.5±1.8	0.008
ERM detachment† (mm)	0.7±1.1	1.5±1.5	0.001
Intraretinal cysts (n [%])	9 (15.2%)	12 (20.0%)	0.632
ERM discontinuity (n [%])	9 (15.2%)	19 (31.7%)	0.032

* after Bonferroni correction a p value <0.005 was considered statistically significant.

† mean ± SD.

## Discussion

As previously shown, the removal of the ILM is beneficial in ERM peeling.[11] However, the ILM persists on the retina in 50.8 to 64% after ERM peeling.[3], [8] Preoperative predictive markers for ILM persistence have not yet been identified. This study shows that a thicker ERM and a loose connection between the ERM and the retina on OCT are associated with ILM persistence in ERM surgery.

Furthermore, our study, which is so far the largest investigating the persistence of the ILM after ERM peeling, confirms incomplete ILM removal in nearly half of the eyes after ERM peeling. To maximize the rate of combined removal we initiated the peeling by grasping the stained ILM at the border of the ERM. Still, our rate of 49.6% was almost identical to the 49.2% reported by Kifuku and coworkers, who had neither performed ICG staining for primary peeling, nor stated any exceptional attention on having primarily grasped the ILM.[3].

This study suggests that preoperative OCT findings can indicate the risk of ILM persistence during surgery. Previous studies have already shown that changes on OCT correlate with preoperative BCVA and are of prognostic value concerning postoperative BCVA.[12] [13].

We found 2 markers on OCT showing statistically significant association with persisting ILM: increased ERM-Th and elevation of the ERM from the retina. This supports the idea that a loose connection between the ERM and the ILM increases the risk of residual ILM after peeling. After histologic evaluation of peeled ERM and ILM, it has been proposed that a remaining lamella of the vitreous cortex after aberrant PVD induces a less adhesive ERM by leaving a thin layer of collagen between the ILM and the ERM.[8] Recent literature demonstrated that areas where the ERM was easy to peel were likely to show elevation of the ERM on OCT, whereas membranes with closer adherence to the retinal surface were more difficult to mobilize. [14] [15] In contrast to the aforementioned study, Kim and coworkers concluded that this space between the ERM and the retinal surface rather represents a tissue free zone than an interlayer of collagen Our findings on OCT are consistent with such a predetermined cleavage plane. It would be interesting to combine preoperative OCT and postoperative histology to shed light on the content of this ERM/ILM interlayer.

The present study also suggests that a thicker ERM increases the risk of ILM persistence. Increased rigidity of thicker membranes could account for a weaker adhesion between the ERM and the ILM. Similarly, it has been proposed that the ERM stiffening after ICG staining facilitates the peeling process of the ERM.[16].

One should note that the application of chemical compounds on the retina risks collateral damage. Nevertheless intraocular staining is currently the gold standard for identifying the ILM during surgery, when used uncritically ICG has been shown to be retinotoxic[9], [10], [16]. The introduction of Tryptane Blue (TB) and later Brillian Blue G (BBG) enhanced the pallet of dyes for surgeons to choose from. [17] [18] Both dies exhibit a better safety profile in vitro and in animal models. [19] TB, however, stains both the ERM and the ILM and is applied under air. BBG is comparable to ICG in its ILM specificity and its ease of use, but shows less discernible contrast than ICG [20], [21]. ICG’s toxicity depends on its concentration, the duration of staining, and the osmolality of the solution. [16] Subsequently, when using ICG meticulous attention towards exposure minimization is warranted.

Still, considering the impossibility of reliable biomicroscopic detection of persisting ILM, especially in case of small remainders, we recommend a second staining in all cases, since we did not find a marker on OCT to definitely predict simultaneous peeling of the ERM and ILM. There are several potential reasons for the later. First, two-dimensional OCT analysis, as performed in this study, might provide insufficient data. Second, the current axial resolution of OCTs is not capable of detailed imaging of the ILM.[22], [23] Concerning the aforementioned issues, further improvement in soft- and hardware of OCTs will provide better resolution and maybe additional algorithms to facilitate a more thorough analysis. This might confine a second staining to preoperatively identified risk groups. However, the sheer absence of a preoperative marker able to definitely predict ILM adherence is possible.

In summary, even when removing the ERM by targeting the ILM, combined removal succeeds in 50% only. A loose connection between the ERM and the retinal surface predisposes for ILM persistence. Preoperative SD-OCT is helpful in identifying risk markers for the persistence of the ILM in ERM surgery.
